# Acute Myeloid Leukemia Relapse Presenting as Complete Monocular Vision Loss due to Optic Nerve Involvement

**DOI:** 10.1155/2016/3794284

**Published:** 2016-09-07

**Authors:** Shyam A. Patel

**Affiliations:** Stanford Cancer Institute, Stanford University Medical Center, 875 Blake Wilbur Drive, Stanford, CA 94305, USA

## Abstract

Acute myeloid leukemia (AML) involvement of the central nervous system is relatively rare, and detection of leptomeningeal disease typically occurs only after a patient presents with neurological symptoms. The case herein describes a 48-year-old man with relapsed/refractory AML of the mixed lineage leukemia rearrangement subtype, who presents with monocular vision loss due to leukemic eye infiltration. MRI revealed right optic nerve sheath enhancement and restricted diffusion concerning for nerve ischemia and infarct from hypercellularity. Cerebrospinal fluid (CSF) analysis showed a total WBC count of 81/mcl with 96% AML blasts. The onset and progression of visual loss were in concordance with rise in peripheral blood blast count. A low threshold for diagnosis of CSF involvement should be maintained in patients with hyperleukocytosis and high-risk cytogenetics so that prompt treatment with whole brain radiation and intrathecal chemotherapy can be delivered. This case suggests that the eye, as an immunoprivileged site, may serve as a sanctuary from which leukemic cells can resurge and contribute to relapsed disease in patients with high-risk cytogenetics.

## 1. Introduction

Acute myeloid leukemia (AML) is a clonal hematopoietic disorder characterized by uncontrolled proliferation of immature myeloid cells. Symptoms of AML at the time of initial diagnosis are reflective of bone marrow replacement of functional erythrocytes, leukocytes, and platelets. Symptoms include fatigue, infections, and bleeding. In some cases, a diagnosis of AML is made after development of symptoms related to distant extramedullary organ involvement. In a recent case of a 57-year-old man with abdominal pain, fever, and new diagnosis of AML, pathology showed leukemic infiltration of the appendix [1]. In a case of a 29-year-old woman with right lower quadrant pain, fever, and vomiting who was diagnosed with intussusception, pathology after hemicolectomy revealed colonic infiltration by leukemic myeloid cells [2].

Within the context of extramedullary involvement, cerebrospinal fluid (CSF) involvement is rare. CSF involvement is more prevalent in patients with myelomonocytic (M4 subtype) or monocytic (M5 subtype) leukemia [3]. Other risk factors include high lactate dehydrogenase (LDH), young age, high-risk cytogenetics, hyperleukocytosis, and African American ethnicity [4–6]. The CSF accounts for approximately 19% of extramedullary relapse (skin and soft tissue are most common) amongst patients who have received allogeneic bone marrow transplant [6]. In patients presenting with altered mental status, headache, visual disturbances, or other neurological symptoms, consideration should be made for leptomeningeal involvement. This is significant because leptomeningeal AML is associated with a poor prognosis and decreased rate of complete response, decreased disease-free survival, and decreased overall survival compared to absence of leptomeningeal disease [4, 7]. The incidence of AML with central nervous system (CNS) involvement is lower than that of acute lymphoblastic leukemia (ALL), and thus routine lumbar puncture is not performed in the absence of neurological symptoms. Disease relapse can sometimes occur from the CNS prior to relapse from the bone marrow [7]. It has been postulated that CNS prophylaxis for AML can improve survival, but this strategy is currently not used given the relatively low incidence of CNS involvement.

Cranial nerve involvement by leukemia can result in a multitude of symptoms, including impaired vision, hearing deficits, vertigo, sensory losses, and others depending on the nerve involved. The most common ocular manifestation of AML is leukemic retinopathy. Other visual manifestations include gaze palsy, which was described in a patient found to have a chloroma of the dorsal pons [8]. In one case report of a 35-year-old woman, the initial presentation of AML consisted of right eye pain, erythema, and swelling, consistent with hypopyon uveitis [9].

## 2. Case Presentation

A 48-year-old man with past medical history of bipolar disorder, hypertension, and gastroesophageal reflux initially presented with several weeks of upper respiratory symptoms, fatigue, syncopal episodes, and right eye vision loss with preservation of only the right lower visual quadrant. His initial labs showed white blood cell (WBC) count of 261,000/mcl with 79% blasts, hemoglobin of 8.8 g/dL, and platelet count of 27,000/mcl. His symptoms were consistent with leukostasis, and he underwent emergency leukapheresis with clear improvement.

Bone marrow aspirate confirmed the diagnosis of AML with the mixed lineage leukemia (MLL) rearrangement with translocation of chromosomes 11 and 19, herein abbreviated t(11;19). He was enrolled into a clinic trial and randomized to receive standard induction with cytarabine and idarubicin. Day 14 bone marrow exam showed hypocellularity, and day 28 bone marrow exam was negative for MLL rearrangements by metaphase analysis indicating that he was in remission. He subsequently underwent cycle 1 of consolidation with high-dose cytarabine with the goal to proceed with allogeneic hematopoietic stem cell transplant (HSCT).

Unfortunately, his disease relapsed following cycle 1 of consolidation with peripheral WBC count of 81,000/mcl with 87% blasts. Repeat bone marrow exam showed 65% involvement of the marrow by leukemic blasts with the MLL rearrangement. A second induction was initiated using lenalidomide followed by mitoxantrone, etoposide, and cytarabine (MEC) with residual disease on day 14. A third induction with MEC only finally achieved hypocellular marrow on day 14.

**Figure 1:**
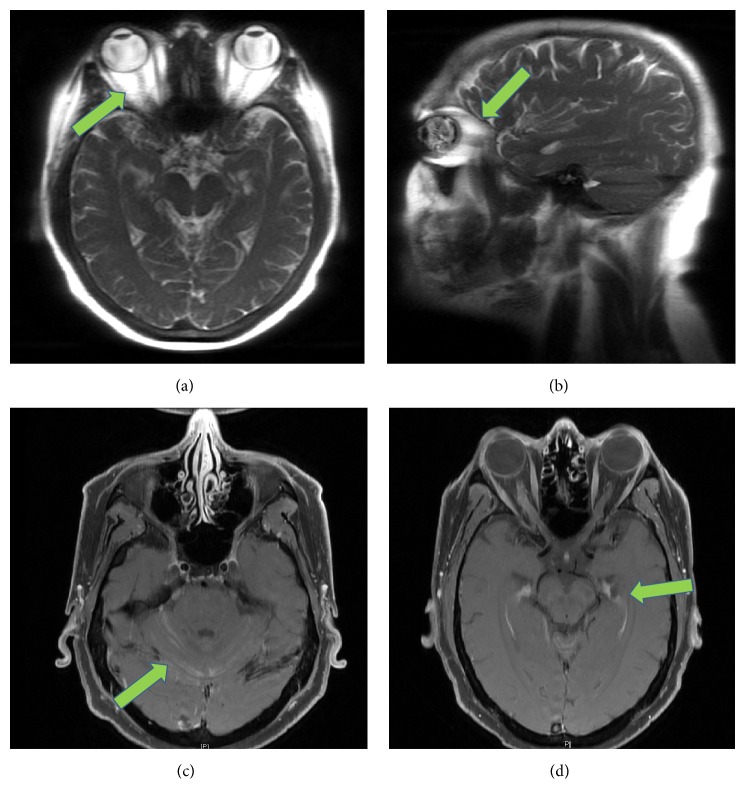
MRI of the brain and orbits. Images show asymmetric enhancement of the right optic nerve sheath (panels (a) and (b)). Arrow denotes greater enhancement in the right optic nerve sheath compared to the left optic nerve sheath, consistent with his clinical presentation. Patient also had bilateral cerebellar surface enhancement (panel (c)) and bilateral trigeminal nerve enhancement (panel (d)).

During the subsequent count recovery period, he developed sudden onset of new pain with extraocular movements of the right eye. Initially, he could only see in the supratentorial fields. Symptoms progressed within a few hours to light perception only in the right eye. Intraocular pressure was 21 mm Hg. Pupillary diameter was 6.5 mm with trace reactivity. Confrontation testing was impaired. Anterior segment exam was normal. Dilated fundoscopic exam was notable for a hyperpigmented area inferonasally with mild overlying vitreous haze but no intraretinal hemorrhages, white spots, or retinitis. There was 3+ edema in the optic nerve. MRI showed enhancement of the right optic nerve sheath, cerebellum, and trigeminal nerve (Figure 1). Flow cytometry of vitreous fluid was negative for AML. Lumbar puncture was performed with CSF showing total WBC count 81/mcl with 96% AML blasts. He promptly received intrathecal cytarabine for relapsed CNS and ocular disease. Visual symptoms were in concordance with the rise in peripheral blood blast count.

Unfortunately, two days after complete vision loss in the right eye, he reported complete vision loss in the left eye with exam concerning for central retinal artery occlusion (CRAO). He was treated with intravenous (IV) acetazolamide, digital manipulation, and anterior chamber paracentesis of the left eye to decrease intraocular pressure. These interventions improved left eye vision temporarily. On the following day, patient developed complete binocular vision loss, severe headache, and vomiting. He was started on high-dose methylprednisolone and underwent repeat MRI, which revealed new restricted diffusion concerning for right optic nerve infarct and persistent right optic nerve enhancement suggestive of neoplastic process, but the left orbit showed no radiographic changes. He was treated with whole brain radiation therapy (WBRT) and therapeutic lumbar puncture with CSF removal plus intrathecal cytarabine, which resulted in resolution of severe headache and nausea. He resumed outpatient WBRT, intrathecal chemotherapy, and a steroid taper. Shortly after completion of WBRT, he was found to have a rising blast count in the peripheral blood that began escalating quickly. He never regained any color or light vision with the above treatments. Due to escalating peripheral blasts counts, he ultimately succumbed to relapsed AML one month later.

## 3. Discussion

Extramedullary involvement by AML is rare, with skin/soft tissue, leptomeningeal, testicular, and ocular involvements being the most common in that respective order [10]. AML with inv(16)(p13.1q22) is known to be associated with extramedullary myeloid sarcoma, whereas AML with t(9;11)(p22q23) has been shown to have association with soft tissue infiltration (gingiva and skin). Risk factors and cytogenetic abnormalities that predispose a patient to develop CNS involvement have not been extensively explored. This area is deserving of interest due to increased difficulties with treatment and the potential for highly morbid associated sequela, such as blindness.

This is the first report of a patient with high-risk cytogenetics who presented with complete monocular vision loss due to involvement of the optic nerve in the absence of radiographic involvement. In a study of the prevalence of ocular changes at the time of AML diagnosis (within two days and prior to initiation of chemotherapy), 35% of patients had ocular lesions, most of which were retinal vascular changes [11]. Others include proptosis and subconjunctival hemorrhage due to thrombocytopenia.

A focal aspect of this case was that imaging (MRI of the brain and orbits) did not reveal clear evidence of leukemic cell infiltration of the left optic nerve, despite bilateral blindness. There have been rare reports of bilateral optic nerve infiltration by leukemia in the absence of radiographic evidence. In the case of a 58-year-old man with chronic myeloid leukemia (CML), MRI showed no optic nerve enhancement and showed normal optic nerve anatomy [12]. Another case of a 64-year-old man with AML with abducens nerve involvement was notable for normal cranial CT and MRI findings, though CSF confirmed malignant cells [13]. This case underscores that a clinical diagnosis of ocular AML should be considered when pathological confirmation on autopsy is not always readily available or feasible.

Secondly, an important distinction to make is the presence of concurrent ocular disorders in the setting of (or associated with) AML compared to ocular disorders caused directly by AML. Several potential etiologies of vision loss include recurrence of retinal hemorrhages due to thrombocytopenia during count recovery, leukemic retinopathy, cytarabine-mediated neurotoxicity, bilateral central retinal artery occlusion by fibrinous emboli, occipital cerebral vascular accident secondary to hypercoagulable state of malignancy, and acute viral or bacterial infection of the CNS. Other possible causes of vision loss include ischemic optic neuropathy (ION). In the case of anterior or posterior ION, one would expect right anterior pupillary defect and altitudinal visual field loss. Patient was unable to undergo retinal angiographic studies, electroretinography (ERG), or visual evoked potentials (VEPs).

The mechanism of extramedullary involvement and relapse is currently unknown. What is also unclear is whether certain molecular features or cytogenetic changes, such as MLL rearrangement (as in this case) or p53 deletion predisposes a subpopulation of AML patients to develop extramedullary disease, particularly in the CNS or eye which can cause major morbidity. In a case of a 23-month-old girl with MLL, leukemic relapse presented as conjunctivitis (intraocular involvement) [14]. The current guidelines do not recommend routine prophylactic intrathecal chemotherapy for patients with AML during induction due to the low rate of involvement at time of diagnosis. However, the incidence and prevalence of ocular involvement by AML are likely underreported, thereby preventing clinicians from determining risk factors that can help better risk stratify patients into categories of likelihood of CNS or ocular involvement. In this case, the patient had two risk factors for CSF involvement, namely, high-risk cytogenetics and hyperleukocytosis on initial presentation. AML patients who have risk factors for CSF involvement should undergo more thorough evaluation for detection of CNS disease and possibly prophylactic intrathecal chemotherapy. Although patients with M4 or M5 classification of AML are more likely to present with CNS relapse, the M2 subtype has been implicated also. Etiology may be related to blood-brain barrier permeability, permitting localization of blasts to the CNS at an early time point [15].

A literature search was performed and notably there were no randomized control trials regarding best management of ocular involvement of AML. Regarding treatment, there are no well-established guidelines to date. One case report described granulocytic sarcoma in the anterior chamber, subconjunctival space, and lacrimal gland as the first sign of AML relapse which preceded medullary relapse by three months [16]. Another case report reported on dyschromatopsia (disturbance of color vision) in a patient with AML, and etiology was thought to involve hypoxia [17]. A recent case report involved a patient with diplopia and strabismus due to abducens nerve involvement from AML, and patient received fludarabine and cytarabine without clinical improvement [13]. One case report of bilateral subfoveal AML reported visual recovery after external beam radiation, and a course of intrathecal cytarabine did not result in improvement [18]. Our patient received intrathecal cytarabine and WBRT with no subsequent improvement in vision. The question remains regarding whether patients with risk factors for CSF involvement would benefit from earlier screening strategies, such as diagnostic lumbar puncture, and prophylactic treatment at an early time point. It is unclear if early treatment or prophylactic treatment of CNS disease can prevent vision loss in AML patients, and there are currently no guidelines for screening or prevention of neurological involvement by AML.

In summary, this case underscores the importance of lowering the threshold for suspicion for leukemic ocular involvement in patients who have known risk factors for CNS involvement, even in the absence of clear radiographic findings, with the goal of early diagnosis which can lead to appropriate treatment such as radiation and intrathecal chemotherapy in a timely manner. This case report contributes to the limited literature that we currently have on ocular AML and highlights the need for better understanding risk factors for extramedullary involvement and for taking preventive steps to avoid devastating morbidity.
